# Akt Regulated Phosphorylation of GSK-3β/Cyclin D1, p21 and p27 Contributes to Cell Proliferation Through Cell Cycle Progression From G1 to S/G2M Phase in Low-Dose Arsenite Exposed HaCat Cells

**DOI:** 10.3389/fphar.2019.01176

**Published:** 2019-10-11

**Authors:** Yao Chen, Xudan Liu, Huanhuan Wang, Shiyi Liu, Nannan Hu, Xin Li

**Affiliations:** Department of Occupational and Environmental Health, Key Laboratory of Arsenic-related Biological Effects and Prevention and Treatment in Liaoning Province, School of Public Health, China Medical University, Shenyang, China

**Keywords:** arsenite, Akt, *p*-GSK-3β, *p*-cyclin D1, *p*-p21, *p*-p27

## Abstract

Arsenic is a toxic environmental contaminant. Long-term exposure to arsenic through drinking water induces human cancers. However, it is as yet uncertain about the mechanisms of arsenic induced carcinogenesis. Although the effects of low-dose arsenicals on proliferation and cell cycle have been revealed by short time exposure, the evidences for long-term exposure were seldom reported. The detailed mechanism has been unclear and supplemented constantly. In the present study, we used normal human keratinocytes (HaCat) to study the effects of long-term, low-dose sodium arsenite (NaAsO_2_) exposure on cell proliferation with emphasis on the Akt regulated cell cycle signaling pathways. Treatment of NaAsO_2_ resulted in increased cell proliferation and promotion of cell cycle progression from G1 to S/G2M phase, both of which could be attenuated by MK2206, a highly selective inhibitor of Akt. Along with the increased expression of phospho-Akt (*p*-Akt, Ser 473), increased expression of *p*-GSK-3β (Ser 9), *p*-p21 (Thr 145), *p*-p27 (Thr 157) and total cyclin D1, and decreased expression of *p*-cyclin D1 (Thr 286), p21 and p27 were also found in the NaAsO_2_ exposed cells. Treatment of MK2206 markedly reversed the expression of all of the above proteins. Our findings indicated that the phosphorylated activation of Akt played a role in the proliferation of HaCat cells upon long-term, low-dose NaAsO_2_ exposure through the phosphorylative regulation of its downstream cell cycle regulating factors of GSK-3β/cyclin D1, p21 and p27, which could induce the promotion of cell cycle progression from G1 to S/G2M phase.

## Introduction

Arsenic is a metalloid widely distributed throughout the earth. The main exposure sources of humans include environment and occupation, as well as medicinal arsenical use. As a natural groundwater contaminant, the main source of human environmental exposure is through consumption of groundwater rich in arsenic. Chronic exposure of arsenic *via* drinking water and its adverse health impacts on humans have been a worldwide health issue in the recent decades (Rahman et al., 2009). It is estimated that nearly 200 million people throughout the world are at risk of toxic exposure to arsenic, nowadays (Hunt et al., 2014). Groundwater used for drinking contaminated by arsenic was first recognized in the 1960s in China and has been a health threat since then. According to a recent report from *Science*, nearly 19.6 million people are reported to be at risk of arsenic-related adverse health injuries through the consumption of arsenic-contaminated groundwater in China (Rodríguez-Lado et al., 2013).

Arsenic is a well-known human carcinogen confirmed by the International Agency for Research on Cancer (IARC). Chronic exposure to arsenic through drinking water has been reported to be associated with cancers including skin, bladder, kidney, liver, and lung (IARC Working Group on the Evaluation of Carcinogenic Risk to Humans, 2004; Celik et al., 2008; Saint-Jacques et al., 2014; Wang W., et al., 2014; Karagas et al., 2015). Among them, skin is believed to be the most sensitive site, therefore, skin cancer is regarded as an early malignancy due to arsenic exposure (Yu et al., 2006). Although high levels of arsenic exposure through drinking water is considered to be associated with the occurrence of arsenic-related cancers, evidences about exposures at relatively lower levels, i.e. less than 100 µg/L, are inconsistent (Cohen et al., 2013). There are increasing evidences suggesting that arsenic-related skin lesions and skin cancers can be induced at levels which are not regarded as harmful previously (Karagas et al., 2015).

Keratinocytes are believed to be the main target cells in arsenic-induced skin carcinogenesis. A concentration-dependent cellular response was observed in keratinocytes exposed to arsenic, which is demonstrated by apoptosis induced by high-dose exposure and proliferation induced by low-dose exposure. Long-term arsenic exposure induced keratinocyte proliferation is possibly involved in the process of skin carcinogenesis (Liao et al., 2011).

Many intracellular signaling pathways were involved in the arsenic induced skin cancer including Akt (Hunt et al., 2014). Akt is a central regulator which play an important role in the regulation of cell metabolism, cell cycle, and apoptosis (Hanada et al., 2004; Sale and Sale, 2008). Relatively high expression or phosphorylation of Akt could be found in many tumor cells including arsenic-related cancers (Carpenter and Jiang, 2013; Cheung and Testa, 2013). Activation of Akt induced by arsenic exposure was also reported in cultured human keratinocytes (Souza et al., 2001). Since the Akt regulated signals are a complicated network system, the detailed signaling pathways involved in arsenic induced carcinogenesis remain to be elucidated.

Although the effects of arsenicals on proliferation and cell cycle have been revealed by short time exposure, the evidences for long-term, low-dose exposure were seldom reported. In the present study, we exposed human keratinocytes (HaCat) to low-dose NaAsO_2_ for 15 weeks and focused on the Akt-regulated cell cycle signals of glycogen synthase kinase 3β (GSK-3β)/cyclin D1, p21^cip1/Waf1^ (p21) and p27^Kip1^ (p27), and discussed the possible role of the phosphorylative activation of Akt and the consequent phosphorylation of its downstream cell cycle regulating factors in the proliferation of low-dose NaAsO_2_ exposed HaCat cells.

## Materials and Methods

### Reagents

Sodium arsenite (NaAsO_2_, ≥90%), dimethyl sulfoxide (DMSO), and ribonuclease A (RNase A) were purchased from Sigma-Aldrich, USA. MK2206 (≥99.91%), a highly selective inhibitor of Akt was purchased from Selleck Chemicals, USA. CellTiter 96 was purchased from Promega, USA. Trypsin, fetal bovine serum (FBS), and minimum essential medium (MEM) were purchased from Thermo Scientific, USA. Protease inhibitor cocktail was purchased from Roche, Germany. Phosphorylase inhibitor P1260 was purchased from Applygen Technologies Inc, Beijing, China. Antibodies specific targeting *p*-GSK-3β (Ser9, sc-81494), *p*-p21 (Thr145, sc-377569), cyclin D1 (sc-246), and β-actin (sc-47778) were purchased from Santa Cruz Biotechnology, USA. Antibodies against *p*-Akt (Ser473, #9271), Akt (#4685S), GSK-3β (#5676), *p*-cyclin D1 (Thr286, #3300), p21 (#2947), p27 (#3686), and MMP9 (#13667) were purchased from Cell Signaling Technology, USA. Antibody of *p*-p27 (Thr157, ab85047) was purchased from Abcam, China.

### Cell Culture and Arsenic Treatment

HaCat was purchased from the cell bank of Chinese Academy of Medical Sciences. Cells were grown in MEM supplemented with 10% FBS and antibiotics in a humidified incubator with 5% CO_2_ at 37°C. An aqueous sterile stock solution of NaAsO_2_ was prepared (1 mmol/L). Stock solution was diluted to the desired final concentration every time before culture media change. A total of three sets of cells were established. Within each set, cells were continuously exposed to NaAsO_2_ at the concentration of 0, 0.05, and 0.1 μmol/L for 15 weeks. After 1 week of the first set was established, the second set was established. On the third week, the third set of cells was established. The cultures were detached with trypsin and transferred to new culture flasks twice a week. Cells were continuously cultured for 15 weeks, then were used for the analysis of cell proliferation, wound-healing, cell cycle, and protein expression for each set of cells.

### Cell Proliferation Assay

For each set of the cells, cell proliferation was analyzed by CellTiter 96 assay. Briefly, HaCat cells were cultured in decuplicate wells for every concentration (0, 0.05 and 0.1 μmol/L); 1×10^4^ viable cells suspended in 200 μl media were added into each well of 96-well plates. The plate was incubated at 37°C with 5% CO_2_. When the monolayer cells grew to approximately 80–85% confluence, MK2206 at the final concentration of 10 μmol/L (dissolved in DMSO) was added into five wells of each concentration. Equal volume of DMSO was added into the other five wells as control. After culturing for another 24 h, media of each well was replaced by 100 μl media containing CellTiter 96 at the concentration of 1:5 (v/v). Cells were then cultured for another 30 minutes, followed by spectrometric measurement under the wavelength of 490 nm. The results were expressed as cell viability relative to the control (proliferation index).

### Wound-Healing Assay

Cells were seeded into 6-well plates and allowed to form confluent monolayers. Cell monolayers were scratched using a 200 μl pipette tip to create a wound and washed once with phosphate-buffered saline (PBS). Fresh MEM and MEM in desired NaAsO_2_ concentration were added into the control wells and NaAsO_2_ treated wells, respectively. MK2206 was added into half of the wells and equal volume of DMSO was added into the other half of the wells. Cells were then cultured for another 48 h. Wound width was monitored over time by microscopy and photographed at 0, 24, and 48 h. The widest and the narrowest widths of each wound were measured. The average width was calculated and regarded as the width of each wound. The final results were obtained from three separate experiments.

### Cell Cycle Assay

Cell preparation was the same with that of the cell proliferation assay. After the treatment of MK2206, cells in the presence or absence of MK2206 were then cultured for another 24 h. The cell cycle was detected by the standard propidium iodide method. Briefly, the treated cells were first trypsinized then fixed with 70% ethanol at 4°C overnight. After being washed twice by PBS, cells were then treated with 100 units/ml RNase A at 37°C for 30 min, followed by staining with 50 mg/ml propidium iodide at 4°C for 30 min with protection from light. The cell cycle proportion was determined by flow cytometric analysis (FACSCanto II system, BD, Franklin Lakes, NJ, USA). The data was analyzed by the software of ModFit LT for Windows Version 3.2, Verity Software House, USA.

### Western Blot Analysis

Cells were seeded into sextuplicate dishes. When the monolayer cells grew to approximately 80-85% confluence, MK2206 at the final concentration of 10 μmol/L (dissolved in DMSO) was added into three dishes. Equal volume of DMSO was added into the other three dishes. After culturing for another 24 h, the treated cells were washed by ice cold PBS twice and extracted with cell lysis buffer (50 mmol/L Tris (pH 8.0), 150 mmol/L NaCl, 0.1% SDS, 1% Nonidet P-40 (NP-40), and 0.5% sodium deoxycholate) supplemented with Roche protease inhibitor cocktail and phosphorylase inhibitor P1260 according to the manufacturer’s instructions. Protein concentrations were measured by a Protein Assay Kit (Bio-Rad, CA, USA) according to the manufacturer’s recommendation. Equal amounts of proteins (30 μg) were separated by 10% sodium dodecyl sulfate polyacrylamide gel electrophoresis and transferred to polyvinylidene difluoride membranes (Millipore Corporation, USA) by electroblotting. Blots were probed with the primary antibodies of Akt (1:500), *p*-Akt (1:500), GSK-3β (1:500), *p*-GSK-3β (1:500), cyclin D1 (1:500), *p*-cyclin D1 (1:500), p27 (1:1000), *p*-p27 (1:500), p21 (1:1000), *p*-p21 (1:500), MMP9 (1:1000), and β-actin (1:2000) at 4°C overnight, followed by incubation with horseradish peroxidase-conjugated secondary antibodies, respectively. Blots were incubated with chemiluminescence reagents and visualized by Electrophoresis Gel Imaging Analysis System (BioSpectrum Imaging System, USA).

### Statistical Analysis

Data were presented as mean ± standard deviation (SD). The differences between the treatment of MK2206(-) and MK2206(+) were analyzed by independent-samples *t* test. The differences of the effects among NaAsO_2_ concentrations (0, 0.05 and 0.1 μmol/L) were analyzed by one-way analysis of variation (ANOVA) followed by Student-Newman-Keuls test or Dunnett’s T3 test depending on whether the variances of the data are equal or not. Statistical evaluation of data was performed by the software of SPSS (version 22.0, Chicago, IL). A *p* value of <0.05 was considered as significant.

## Results

### Repeated Low-Dose NaAsO_2_ Exposure Leaded to HaCat Cell Proliferation

**Figure 1:**
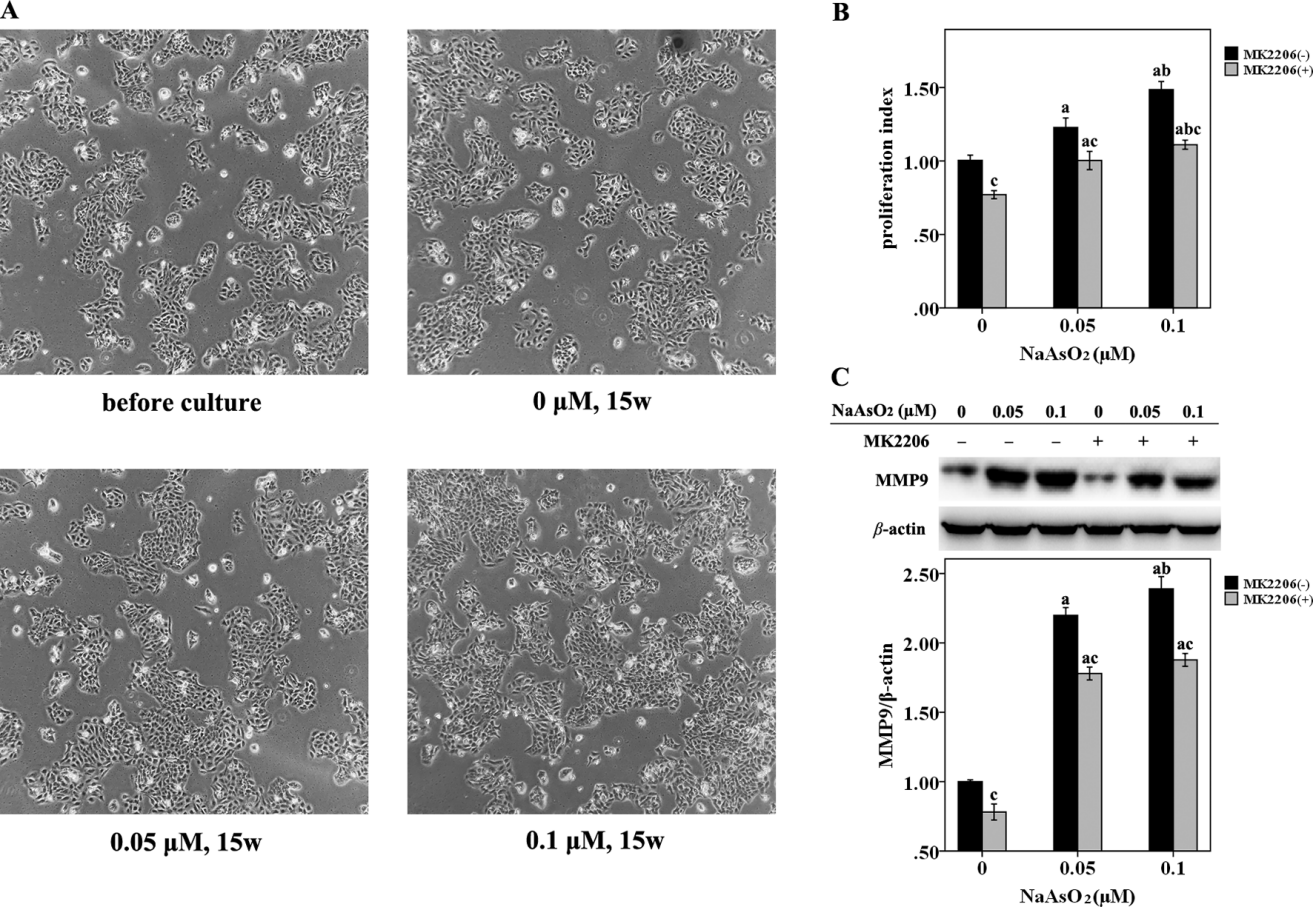
Repeated low-dose NaAsO_2_ exposure resulted in increased proliferative capability and MMP9 expression in HaCat cell. Cells were continuously exposed to NaAsO_2_ for 15 weeks at the concentration of 0, 0.05, and 0.1 μmol/L. A total of three sets of cells were established. **(A)** Cell photos taken before long-term culture and after culture for 15 weeks. No morphological alterations were observed in the NaAsO_2_ exposed cells. **(B)** For each set of the cell, cell proliferation was analyzed by CellTiter 96 assay. Similar results were obtained from the three sets of cells. A representative figure was presented. The NaAsO_2_ exposed cells showed increased proliferative capability, which could be attenuated by MK2206 (10 μmol/L, 24 h). **(C)** The expression of MMP9 was analyzed by Western Blot assay. Long-term NaAsO_2_ exposure resulted in increased expressions of MMP9 in the HaCat cells, which could be attenuated by the treatment of MK2206 (10 μmol/L, 24 h). Significant difference was defined as *p* less than 0.05. a, vs. the corresponding 0 μM group; b, vs. the corresponding 0.05 μM group; c, vs. the MK2206(-) group of the same NaAsO_2_ concentration.

HaCat cells were repeatedly exposed to NaAsO_2_ at different concentrations (0, 0.05 and 0.1 μmol/L) for 15 weeks. No morphological alterations were observed in the NaAsO_2_ exposed cells. The cells looked the same in both the size and shape with those before chronic culture (**Figure 1A**). The NaAsO_2_ exposed cells showed an increased proliferative capability while MK2206, a highly selective inhibitor of Akt, significantly decreased the proliferation of NaAsO_2_ exposed cells (**Figure 1B**). At the same time, MMP 9, one of the matrix metalloproteinases which is abnormally abundant in the microenvironment during carcinogenesis, was found significantly increased in the NaAsO_2_ exposed cells. Treatment of MK2206 attenuated the level of MMP9 which indicated the role of Akt in regulating MMP9 activation (**Figure 1C**).

**Figure 2:**
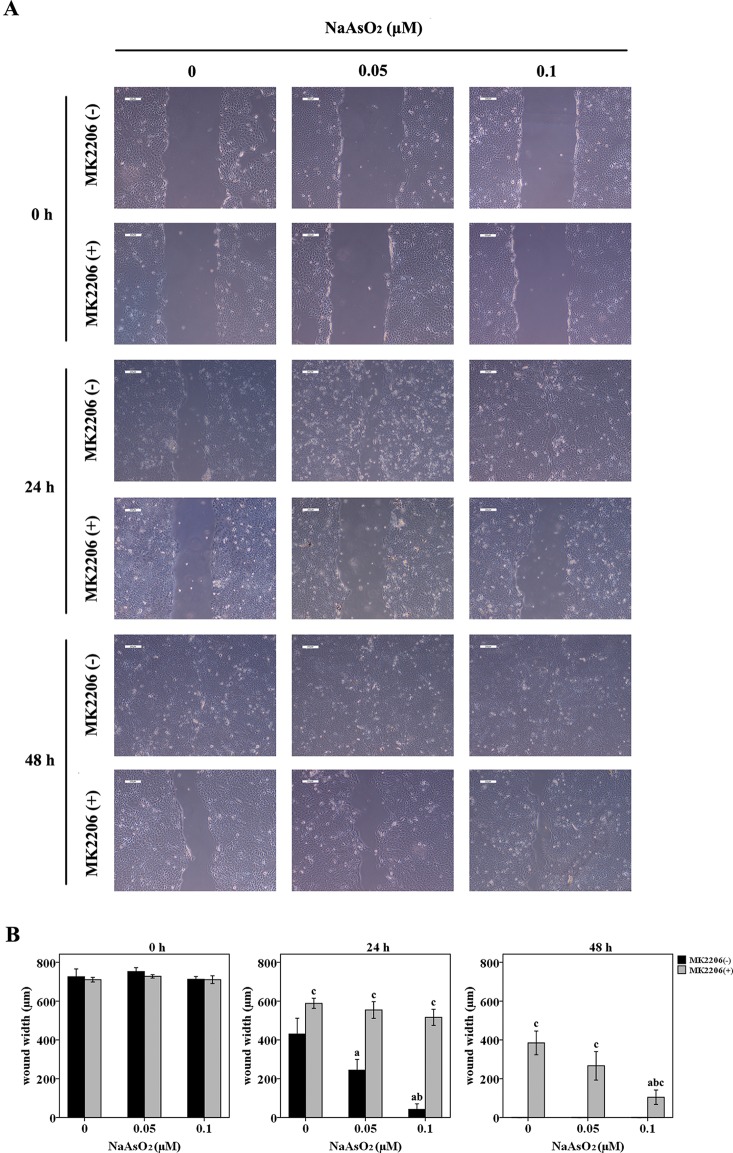
Repeated low-dose NaAsO_2_ exposure increased the wound closure speed of HaCat cells. Cells were seeded into 6-well plates. The cell monolayers were scratched using a 200 μl pipette tip to create a wound. Half of the cells were treated with MK2206 (10 μmol/L). DMSO was added into the other half of the cells. Wound width was monitored over time by microscopy and photographed immediately at the time point of 0, 24 and 48 h after MK2206 treatment. **(A)** Images are the representative photos of three separate experiments. **(B)** Quantitation of wound width (*n* = 3). NaAsO_2_ exposure increased the wound closure speed, no matter in the presence or absence of MK2206. Although wound closure was inhibited by the treatment of MK2206, NaAsO_2_ exposed cells showed higher wound-healing capability than that of the control cells. Significant difference was defined as *p* less than 0.05. a, vs. the corresponding 0 μM group; b, vs. the corresponding 0.05 μM group; c, vs. the MK2206(-) group of the same NaAsO_2_ concentration.

The wound-healing assay revealed that NaAsO_2_ exposure increased the wound closure speed after a 24-h incubation. The higher the NaAsO_2_ concentration, the higher the wound recovery speed (**Figure 2A**, line 3; **Figure 2B**). However, NaAsO_2_ induced increased wound closure was inhibited by the treatment of MK2206 (**Figure 2A**, line 4; **Figure 2B**). At the time point of 48 h, all the wounds of cells without MK2206 treatment were closed since the culture time was long enough for wound healing (**Figure 2A**, line 5). Although wound closure was still inhibited by MK2206, NaAsO_2_ exposed cells showed higher wound-healing capability than that of the control cells (**Figure 2B**). Cells of the 0.1μmol/L group showed the highest wound-healing capability (**Figure 2A**, line 6). These results indicated that repeated low-dose NaAsO_2_ exposure promoted the proliferation of HaCat cell and that Akt, a central player in the regulation of various physiological functions including cell cycle, was involved in the proliferation induced by low-dose NaAsO_2_ exposure.

### Repeated Low-Dose NaAsO_2_ Exposure Promoted Cell Cycle Progression From G1 to S/G2M Phase

**Figure 3:**
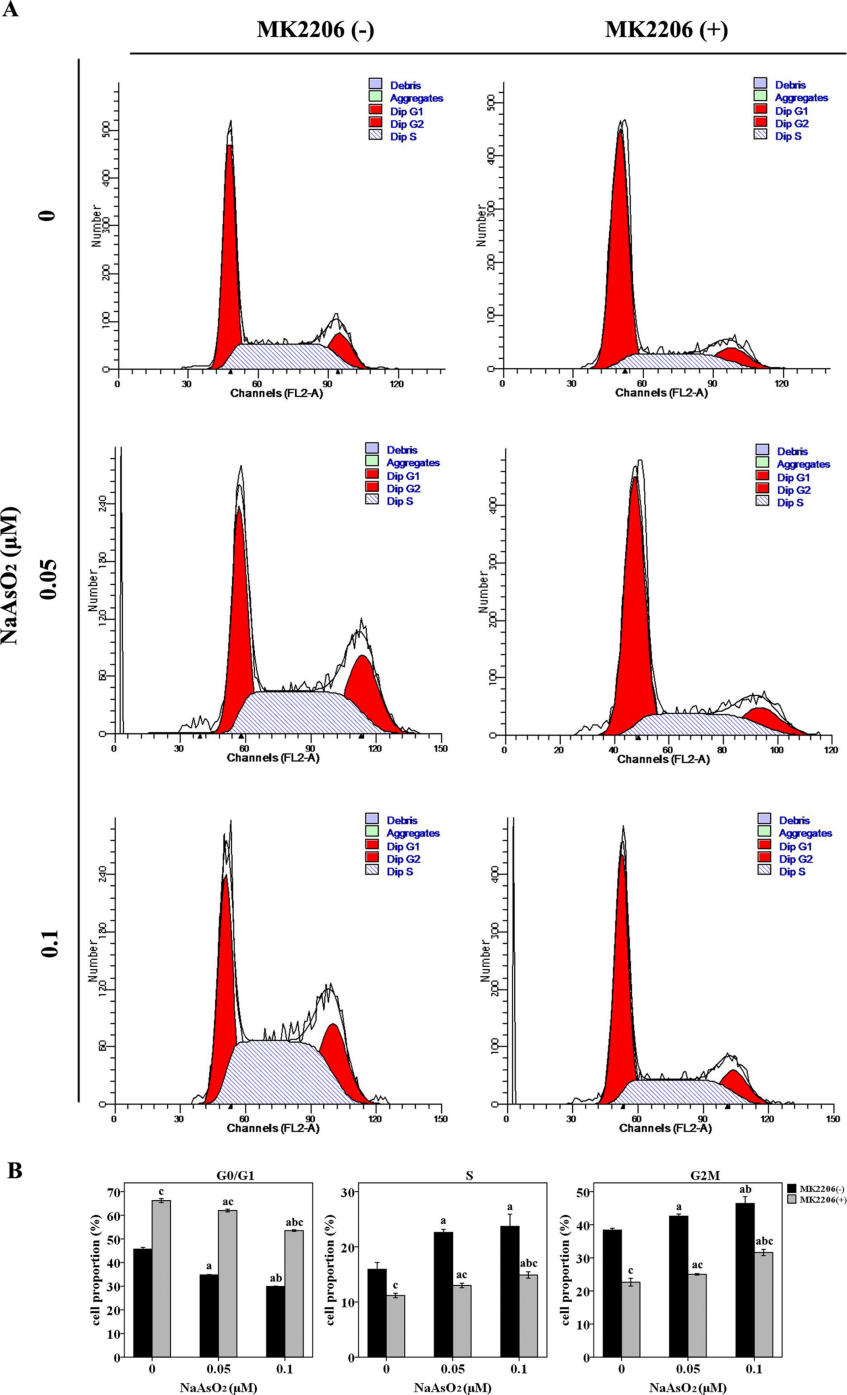
Repeated low-dose NaAsO_2_ exposure promoted HaCat cell cycle progression from G0/G1 to S/G2M phase. Cell cycle was detected by the standard propidium iodide method. **(A)** Cell cycle analysis evaluated by flow cytometry. Images are the representative results of three separate experiments. **(B)** Quantitative graphs of the cell proportions divided by cell cycle (*n* = 3). NaAsO_2_ exposure resulted in decreased proportion of G0/G1 phase, but increased proportions of S and G2M phases, no matter in the presence or absence of MK2206. NaAsO_2_ induced cell cycle progression from G0/G1 to S/G2M phase could be attenuated by the treatment of MK2206. Significant difference was defined as *p* less than 0.05. a, vs. the corresponding 0 μM group; b, vs. the corresponding 0.05 μM group; c, vs. the MK2206(-) group of the same NaAsO_2_ concentration.

Since cell cycle regulation plays an important role in the induction of cell proliferation, we further analyzed the cell cycle distribution in this study. NaAsO_2_ exposure induced significantly decreased cell proportions of G0/G1 phase and increased cell proportions of both S and G2M phase (**Figure 3**). This finding suggested that low-dose NaAsO_2_ exposure promoted cell cycle progression from G1 to S/G2M phase, which might explain the increased proliferation in NaAsO_2_ exposed HaCat cells. Inversely, treatment of MK2206 increased the cell proportions of G0/G1 phase and decreased the cell proportions of both S and G2M phases in the NaAsO_2_ exposed cells (**Figure 3**), which suggested that cell cycle progression from G1 to S/G2M phase could be abolished by Akt inhibition. This result indicated that Akt played a role in the cell cycle regulation of NaAsO_2_ exposed HaCat cell.

### Repeated Low-Dose NaAsO_2_ Induced Phosphoraled Protein Expression of GSK-3β/CyclinD1, p21, and p27 Was Regulated by Akt

**Figure 4:**
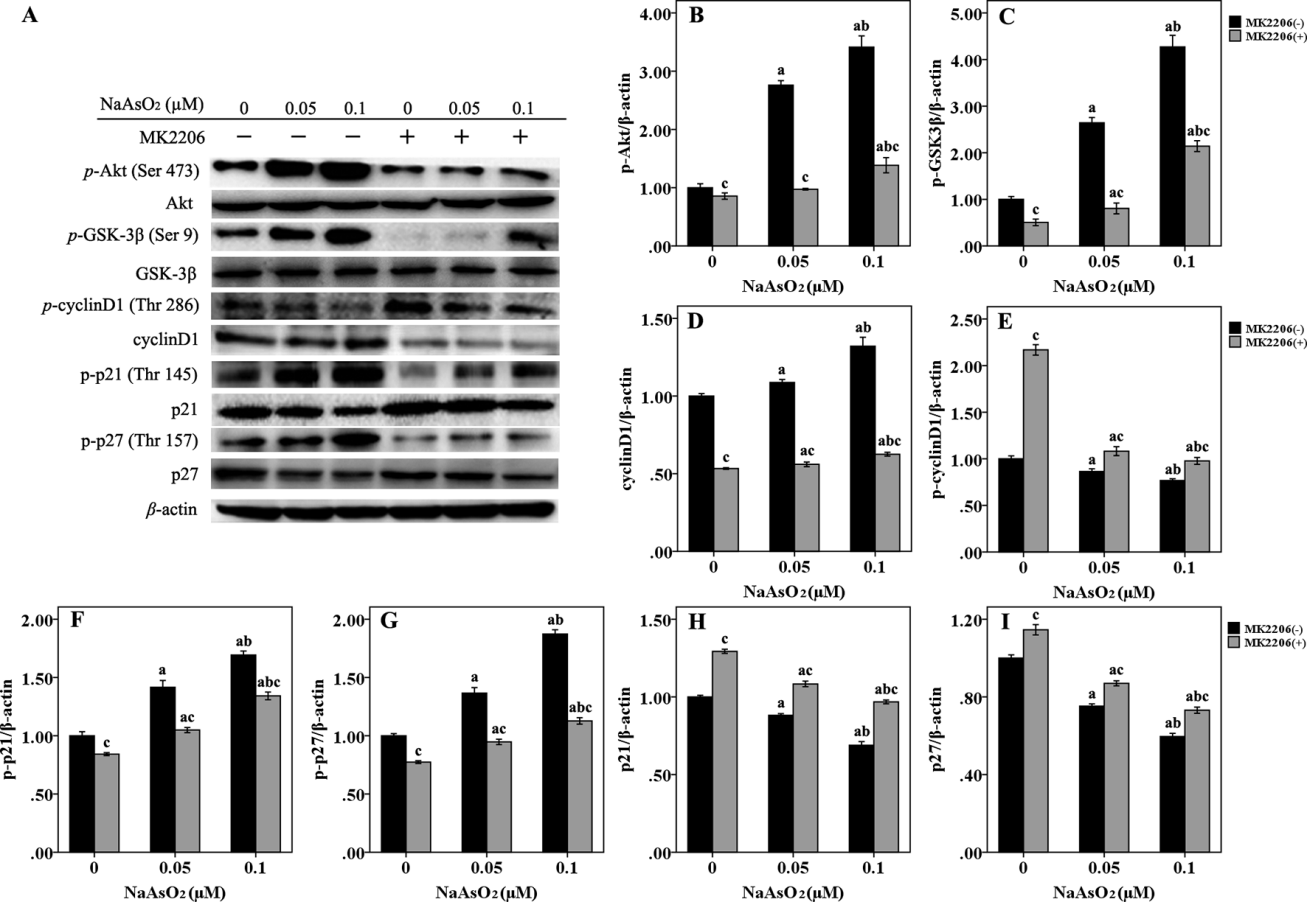
Repeated low-dose NaAsO_2_ exposure induced phosphorylated protein expression of GSK-3β/cyclinD1, p21 and p27 was regulated by AKT. Protein expression was detected by the Western blot analysis. **(A)** Images are the representative results of three separate experiments. Quantitative graphs (*n* = 3) showed the relative intensity of the target proteins compare with β-actin, including *p*-AKT **(B)**, *p*-GSK-3β **(C)**, total cyclin D1 **(D)**, *p*-cyclin D1 **(E)**, *p*-p21 **(F)**, *p*-p27 **(G)**, total p21 **(H)** and total p27 **(I)**. NaAsO_2_ exposure induced increased expression of *p*-AKT, *p*-GSK-3β, total cyclin D1, *p*-p21, and *p*-p27, but decreased expression of *p*-cyclin D1, total p21, and total p27. Treatment of MK2206 significantly reversed the expression of all of the above proteins. Significant difference was defined as *p* less than 0.05. a, vs. the corresponding 0 μM group; b, vs. the corresponding 0.05 μM group; c, vs. the MK2206(-) group of the same NaAsO_2_ concentration.

In order to further elucidate the mechanism of cell cycle progression, we focused on Akt and its downstream factors of GSK-3β/cyclinD1, p21, and p27, which were associated with cell cycle regulation (**Figure 4A**). Results of western blot analysis indicated that Akt could be fully activated by both of the two concentrations of NaAsO_2_ through phosphorylation at Ser 473. The phosphorylated activation could be attenuated by the treatment of MK2206 (**Figure 4B**). Along with the increase of *p*-Akt expression, GSK-3β, one of the downstream factors of Akt, was found to be significantly phosphorylated at the Ser 9 site (**Figure 4C**). The expression of total cyclin D1 was also increased in the NaAsO_2_ exposed cells (**Figure 4D**), while the expression of *p*-cyclin D1 (Thr 286) was markedly decreased (**Figure 4E**). Treatment of MK2206 decreased the expression of *p*-GSK-3β (Ser 9) and total cyclin D1, but increased the expression of *p*-cyclin D1 (Thr 286) in the NaAsO_2_ exposed cells. Since cyclin D1 is one of the downstream factors of GSK-3β and phosphorylation of GSK-3β at Ser 9 results in the inactivation of its kinase capability which will lead to the decreased phosphorylation of cyclin D1 and the consequent increased level of total cyclin D1, our findings indicated that signaling pathway of Akt/GSK-3β/cyclin D1 was involved in the cell cycle regulation of NaAsO_2_ exposed HaCat cells.

p21 and p27, two main members of negative regulators of cell cycle, are also downstream factors of Akt, which are phosphorylated by Akt at Thr 145 and Thr 157, respectively. In the present study, along with the increased expression of *p*-Akt, the expression of both *p*-p21 (Thr 145) (**Figure 4F**) and *p*-p27 (Thr 157) (**Figure 4G**) was significantly increased in the NaAsO_2_ exposed cells. At the same time, the expression of total p21 (**Figure 4H**) and p27 (**Figure 4I**) was found to be decreased. Treatment of MK2206 markedly decreased the expression of both of the two phosphorylated proteins, but increased the expression of their total proteins. Since phosphorylation of these cell cycle inhibitors will lead to the cytoplasmic retention of these proteins and precluding their binding and inhibition of the cyclin/CDK complexes, our results suggested that the Akt regulated phosphorylation of p21 and p27 were also involved in the promotion of cell cycle progression from G1 to S/G2M phase in the NaAsO_2_ exposed HaCat cells.

## Discussion

Arsenic has been classified as a group 1 carcinogen by IARC. Although various hypotheses were proposed, it is as yet uncertain about the mechanism of arsenic induced carcinogenesis. Due to the weak mutagenic capability of arsenic, it is considered that the activation of signaling pathways and gene expression which play a role in the cell growth may function in the process of carcinogenesis (Bernstam and Nriagu, 2000). Some recent studies suggested that Akt signals which play an important role in cellular proliferation and survival were also involved in arsenic induced carcinogenesis (Carpenter and Jiang, 2013). Since much of the findings about Akt signals were obtained from the studies of acute arsenic exposure at relatively higher concentrations, studies about the effects of long-term exposure at lower dose is needed, considering the emerging facts that arsenic may increase the risk of cancers at lower levels that is not regarded as harmful before (Karagas et al., 2015; Carlin et al., 2016). Skin is the primary target organ of arsenic (Centeno et al., 2002). In addition, the main type of arsenic-related skin cancers is squamous cell carcinomas arising in keratosis (Yeh et al., 1968; Hunt et al., 2014). Therefore, we investigated the effects of long-term, low-dose arsenic exposure on Akt signals, particularly on its downstream factors participating in cell cycle regulation in human keratinocytes.

Several studies previously reported the effects of repeated low-dose arsenic exposure in HaCat cells. For example, Ouyang et al. (2008) found that repeated NaAsO_2_ exposure (2.5 μM, 8 weeks) could induce the increased anchorage independent growth capacity in HaCat cells. Al-Eryani et al. (2017) reported that NaAsO_2_ exposure (0.1 μM, 7 weeks) resulted in differential gene expression which indicated the dysregulation of cell cycle control. Liu et al. (2015) proved that apart from the increase of cellular proliferation, low-dose As_2_O_3_ (0.1 and 0.2 μM, 4 weeks) exposure could also induce the malignant transformation of HaCat cells such as epithelial-to-mesenchymal transition, matrix metalloproteinases activation, and anchorage-independent growth.

The high concentration of NaAsO_2_ used in our study is 0.1μmol/L, which is comparable with the average blood arsenic levels of patients in Inner Mongolia, China where arsenic-related skin lesions are common (Pi et al., 2000). Cell proliferation and promotion of cell cycle progression from G1 to S/G2M phase could be induced not only by the high concentration, but also by the low concentration of 0.05 μmol/L in the present study. To our knowledge, it is the lowest concentration that have been reported up to now which could induce cell proliferation and cell cycle change upon repeated low-dose arsenic exposure.

MK2206 is an allosteric Akt kinase inhibitor. It binds to Akt and induces a conformational change in the Akt protein to the ‘closed’ cytoplasmic conformation which prevent its membrane translocation and phosphorylation that are necessary for its activation (Lindsley, 2010; Song et al., 2019). In this study, cell proliferation, cell cycle progression, and phosphorylated activation of Akt induced by NaAsO_2_ exposure could be attenuated by MK2206. These findings indicated that the phosphorylated activation of Akt and its consequent regulation through cell cycle were at least one of the reasons involved in the low-dose NaAsO_2_ induced cell proliferation.

Cyclin D1, which regulates the G1/S point of the cell cycle, is reported to be over expressed in many tumors (Casimiro et al., 2014). Overexpression of total cyclin D1 might be a consistent event during the carcinogenesis induced by arsenic exposure. It has been reported that signaling pathways including c-Jun/AP-1 (Zhang et al., 2009), p38 MAPK (Liu et al., 2010), JNK/c-Jun (Li et al., 2011), and Erk1/2 (Liu et al., 2010; Huang et al., 2011) can be activated upon NaAsO_2_ exposure which results in the overexpression of total cyclin D1.

Activation of Akt signal was also reported to directly induce the overexpression of total cyclin D1 in several cell lines exposed to low-dose arsenicals within 12 to 48 h. For example, Liu et al. (2010) reported that inhibiting the activation of Akt decreased the low-dose As_2_O_3_ (0.1 μmol/L, 24 h) induced expression of total cyclin D1 in human breast epithelial cells (MCF10A). Ouyang et al. (2006) proved that low-dose NaAsO_2_ (5 μmol/L, 18, 30 and 48 h) induced overexpression of total cyclin D1 could be attenuated by the Akt inhibitors in mouse epidermal Cl41 cells. The group of Ouyang (2007; 2008) also provided direct evidences that Akt played a role in the overexpression of total cyclin D1 in HaCat cells and normal human epidermal keratinocytes (NHEKs) upon low-dose NaAsO_2_ exposure (0.15-5 μmol/L, 12-48 h).

Although the relationship between Akt activation and total cyclin D1 overexpression upon low-dose arsenic treatment was confirmed by short time exposure, the evidences for long-term exposure were seldom reported. In our study, we continuously exposed HaCat cells by NaAsO_2_ for 15 weeks under the concentrations of 0.05 and 0.1 μmol/L, and found that NaAsO_2_ induced overexpression of total cyclin D1 could be attenuated by MK2206. This result indicated that Akt was involved in the regulation of cyclin D1. In order to further reveal the linkage between Akt activation and cyclin D1 overexpression, we focused on the potential regulator of GSK-3β, which is one of the downstream factors of Akt.

Recent reports indicated that Akt played a role in cell cycle progression partially by the phosphorylation at Ser 9 of GSK-3β, which resulted in the inhibition of GSK-3β activity. While GSK-3β is a kinase responsible for the phosphorylation of cyclin D1 that results in its degradation (Cheung and Testa, 2013; Song et al., 2019), the phosphorylation of cyclin D1 at Thr 286 by GSK-3β could trigger nuclear-cytoplasmic translocation of cyclin D1, which could result in the ubiquitin mediated proteolytic degradation of cyclin D1 in the cytoplasm (Shimura, 2011).

In the present study, apart from the increased expression of *p*-Akt and total cyclin D1, we further found the corresponding increased expression of *p*-GSK-3β (Ser 9) and decreased expression of *p*-cyclin D1 (Thr 286). Treatment of MK2206 resulted in the decreased expression of *p*-Akt along with decreased expression of *p*-GSK-3β (Ser 9), increased expression of *p*-cyclin D1 (Thr 286), and decreased expression of total cyclin D1. Since the activity of GSK-3β is inhibited by Akt-dependent phophorylation at Ser 9 and GSK-3β mediated cyclin D1 protein degradation through phophorylating it at Thr 286, our findings suggested that the activation of Akt/GSK-3β signaling pathway was involved in the overexpression of cyclin D1, which might contribute to the cell cycle progression upon low-dose NaAsO_2_ exposure.

Cell cycle is mainly regulated by cyclins and the cyclin dependent kinases (CDKs). The cyclin inhibitor protein/kinase inhibitor protein (Cip/Kip) family has the capability of inhibiting cyclin/CDK complex. p21 and p27 are two main members of Cip/Kip family, both of which could regulate the G1 check point of the cell cycle through binding to CDK/cyclin complexes (Starostina and Kipreos, 2012). The expression of p21 was reported to be decreased in HaCat cells upon long-term, low-dose NaAsO_2_ exposure, and dysfunction of p53 was suggested to be a possible mechanism (Komissarova and Rossman, 2010; Li et al., 2010). Decreased expression of p27 was also reported in cells chronically exposed to NaAsO_2_ which contributed to the proliferation of murine fibroblast cells (Trouba et al., 2000).

It has been suggested that the cell cycle inhibitors, p21 and p27, could be phosphorylated at Thr 145 and Thr 157, respectively, by Akt, which will lead to cytoplasmic retention of both of the proteins and finally prevent their binding and inhibition of the cyclin/CDK complexes (Cheung and Testa, 2013). In the present study, along with the decreased expressions of p21 and p27, we also found significantly increased expressions of *p*-p21 (Thr 145) and *p*-p27 (Thr 157) in the NaAsO_2_ exposed cells. The expression of both *p*-p21 (Thr 145) and *p*-p27 (Thr 157) could be attenuated by the treatment of MK2206. This finding indicated that the phosphorylation of p21 and p27 regulated by Akt also played a role in the cell cycle progression induced by low-dose NaAsO_2_ exposure.

In our study, along with the increased proliferative capability and promotion of cell cycle progression, we also found increased expressions of MMP9 in the NaAsO_2_ exposed HaCat cells. MMP9 is one of the members of Matrix metalloproteinases (MMPs). MMPs are important mediators of alterations found in the microenvironment during carcinogenesis, which play an important role in extracellular matrix degradation, migration, and invasion. According to the recent reports, MMPs also play a role in regulating initial steps of carcinogenesis (Yadav et al., 2014). It has been reported that the activation of MMP9 contributes to the low-dose arsenic induced malignant transformation of HaCat cells (Pi et al., 2008). Treatment of MK2206 attenuated the expression of MMP9 in the NaAsO_2_ exposed cells of this study. This result indicated the role of Akt in regulating MMP9 activation in the long-term, low-dose NaAsO_2_ exposed HaCat cells. Some other reports also provided evidences of Akt-regulated MMP9 in the carcinogenesis (Wang F., et al., 2014; Cheng et al., 2019).

Finally, using data from only one transformed cell line (HaCat Keratinocytes) does not justify the overall effects of low dose NaAsO_2_ exposure on cell proliferation mediated by Akt signaling. This limited the findings of this study.

## Conclusions

Taken together, our results suggested that the phosphorylative activation of Akt and its consequent phosphorylative regulation of its downstream cell cycle regulating factors of GSK-3β/cyclinD1, p21, and p27 played a role in the cell proliferation through the promotion of cell cycle progression from G1 to S/G2M phase in long-term, low-dose NaAsO_2_ exposed HaCat cells.

## Data Availability Statement

The datasets generated for this study are available on request to the corresponding author.

## Author Contributions

YC, XLiu, HW, SL, and NH carried out the experiments. YC drafted the manuscript. XLi conceived, designed, and supervised the experiments, and edited the manuscript. All authors contributed to revision and approved the manuscript.

## Funding

This work was supported by the National Natural Science Foundation of China (NSFC) [grant number 81872568] and the Natural Science Foundation of Liaoning Province of China [grant number 20180550322].

## Conflict of Interest

The authors declare that the research was conducted in the absence of any commercial or financial relationships that could be construed as a potential conflict of interest.
